# Predictors of Mortality in Neonates and Infants Hospitalized With Sepsis or Serious Infections in Developing Countries: A Systematic Review

**DOI:** 10.3389/fped.2018.00277

**Published:** 2018-10-04

**Authors:** Li(Danny) Liang, Naima Kotadia, Lacey English, Niranjan Kissoon, J. Mark Ansermino, Jerome Kabakyenga, Pascal M. Lavoie, Matthew O. Wiens

**Affiliations:** ^1^Faculty of Medicine, University of Toronto, Toronto, ON, Canada; ^2^Faculty of Medicine, University of British Columbia, Vancouver, BC, Canada; ^3^School of Medicine, University of North Carolina, Chapel Hill, NC, United States; ^4^Department of Pediatrics, University of British Columbia, Vancouver, BC, Canada; ^5^Center for International Child Health, British Columbia Children's Hospital, Vancouver, BC, Canada; ^6^Department of Anesthesiology, Pharmacology and Therapeutics, Faculty of Medicine, University of British Columbia, Vancouver, BC, Canada; ^7^Department of Anesthesia, British Columbia Children's Hospital and University of British Columbia, Vancouver, BC, Canada; ^8^Maternal Newborn and Child Health Institute, Mbarara University of Science and Technology, Mbarara, Uganda; ^9^Faculty of Medicine, Mbarara University of Science and Technology, Mbarara, Uganda; ^10^Division of Neonatology, Department of Pediatrics, University of British Columbia, Vancouver, BC, Canada

**Keywords:** risk prediction, neonatal mortality, infant mortality, systematic review, developing countries, infectious disease, sepsis, hospital mortality

## Abstract

**Background:** Neonates and infants comprise the majority of the 6 million annual deaths under 5 years of age around the world. Most of these deaths occur in low/middle income countries (LMICs) and are preventable. However, the clinical identification of neonates and infants at imminent risk of death is challenging in developing countries.

**Objective:** To systematically review the literature on clinical risk factors for mortality in infants under 12 months of age hospitalized for sepsis or serious infections in LMICs.

**Methods:** MEDLINE and EMBASE were systematically searched using MeSH terms through April 2017. Abstracts were independently screened by two reviewers. Subsequently, full-text articles were selected by two independent reviewers based on PICOS criteria for inclusion in the final analysis. Study data were qualitatively synthesized without quantitative pooling of data due to heterogeneity in study populations and methodology.

**Results:** A total of 1,139 abstracts were screened, and 169 full-text articles were selected for text review. Of these, 45 articles were included in the analysis, with 21 articles featuring neonatal populations (under 28 days of age) exclusively. Most studies were from Sub-Saharan Africa and South Asia. Risk factors for mortality varied significantly according to study populations. For neonatal deaths, prematurity, low birth-weight and young age at presentation were most frequently associated with mortality. For infant deaths, malnutrition, lack of breastfeeding and low oxygen saturation were associated with mortality in the highest number of studies.

**Conclusions:** Risk factors for mortality differ between the neonatal and young infant age groups and were also dependant on the study population. These data can serve as a starting point for the development of individualized predictive models for in-hospital and post-discharge mortality and for the development of interventions to improve outcomes among these high-risk groups.

## Introduction

Significant progresses have been made over the past two decades in reducing global under-5 mortality from 91 deaths per 1,000 live births in 1990 to 43 deaths per 1,000 live births in 2015 (1). However, mortality in neonates (under 28 days of age) and infants (under 1 year of age) remains disproportionately high, representing over two-thirds of under-5 deaths in children below 5 years of age (2). Of these deaths, 90% occur in developing countries (3), with the highest neonatal mortality rates occurring in Sub-Saharan Africa. Thus, interventions among neonates and infants are urgently needed in these countries if the recent UN Sustainable Development Goals are to be achieved, targeting to decrease under-5 mortality to < 25 per 1,000 live births and neonatal mortality to < 12 per 1,000 live births by 2030 (4, 5).

Reducing mortality in neonates and young infants has lagged significantly behind that of older pediatric populations (6). Although no consensus has been made, some studies suggest preterm birth, intrapartum complications, and sepsis are leading causes of death among neonates (7). The relative risk of death from these events ranges from 10 to 36 times greater in LMICs, as compared to high income countries (3).

A population-based health survey in 56 countries from 1990 to 2002 identified main factors contributing to infant mortality around the world, including first births, shorter birth interval, male sex, gestational multiplicity, and rural setting (8, 9). Infants of mothers with less education or who lived further away from health care resources were also at greater risk of death (8, 9).

However, in spite of this knowledge, our ability to develop effective interventions for this age group remains limited, as causes of death are multi-factorial and often involve a greater social context. Published systematic reviews have attempted to discern more age-specific factors, but these studies have largely focused on high resource settings (10). Given that the greatest burden of neonatal and infant deaths occur in LMICs, this is an important limitation (11). Finally, underserved populations tend to have limited research, making the benefits of systematic reviews in the identification of gaps in these cases all the more impactful (12, 13).

The objective of this systematic review was to develop an evidence base of studies assessing risk factors for mortality among newborns and infants who are hospitalized in LMICs. The focus of this literature review was serious infections and sepsis, as they are the most commonly identified, and potentially highly preventable (14), causes of neonatal and infant deaths in LMICs.

## Methods

### Study eligibility criteria and systematic search

This systematic review focused on children < 1 year old evaluated for sepsis or other serious infections in LMICs. Study eligibility was defined according to the conventional Populations, Interventions, Comparators, Outcomes, and Study Design (PICOS) criteria, determined *a priori* (Table 1).

**Table 1:** PICOS criteria outlining study eligibility.

**Criteria**	**Inclusion**
**Population**	Newborns and young infants hospitalized with sepsis or other serious infection in a resource poor/developing country (majority of patients must be < 1 year of age, or a multivariate analysis adjusting for age must be conducted) •Studies conducted in developed countries will be excluded •Studies involving non-admitted patients (ex. Ambulatory or community) or surgical patients will be excluded
**Interventions**	Usual care, or care during an intervention of any kind^*^
**Comparisons**	N/A
**Outcomes**	Studies must evaluate and report risk factors, along with a risk estimate (OR, RR, etc) or valid statistical analysis for at least one of the following outcomes:
	•In-hospital mortality prior to discharge
	•Post-discharge mortality occurring either in community or upon readmission
**Study designs**	Studies must be one of the following:
	•Single arm or multi-arm trials
	•Prospective cohort study
	•Retrospective cohort studies
	•One case-control study
**Language**	English language only

* *Please note that in all studies included except one, the intervention was usual care. If any additional interventions were employed and the results showed statistical significance, the relative risks for the control arm as well as the intervention arm will be described separately in the results section*.

A study was included if (i) it presented original data from either a prospective or retrospective cohort study or from a randomized controlled trial, (ii) the majority of the subjects were under 1 year of age or a multivariate analysis adjusting for age was conducted, or a subgroup analysis for specific mortality risk factors was collected for patients < 1 year old, (iii) the study was conducted in a developing country (defined as countries classified by the United Nations Development Program (UNDP) in its 2016 report as having a low or medium Human Development Index (15)), and (iv) published in English.

Studies were excluded if (i) some or all of the patients were not admitted (ex. ambulatory or community health facilities), (ii) it represented a surgical population since mortality risk factors for surgeries would likely differ from those of acute illness, or (iii) the study population included nosocomial infections (Table 1).

MEDLINE and EMBASE databases were searched with the assistance of a medical librarian (Appendix 1 and Appendix 2), using the Ovid platform. The search dates were from database inception to April 2017.

### Study selection and data extraction

Two investigators (LL and NK) independently conducted two rounds of review to determine study eligibility among identified articles. Articles were first screened based on the abstract using the PICOS criteria defined in Table 1, where articles clearly meeting exclusion criteria were immediately discarded. A second round of review involved screening the remaining manuscripts in full text, to determine final eligibility. At both stages, discrepancies were resolved by consensus or by arbitration by a third investigator (MW).

For all included articles in the analysis, the following study characteristics were extracted, including: country/region, study period, study design, study population, number of subjects, number of deaths, proportion of participants under 12 months and under 1 month of age, type of analyses conducted for risk factors (i.e., multivariate vs. univariate) and length of post-discharge follow-up. All variables included in the mortality risk factor analyses were recorded. If both univariate and multivariate analyses were performed, only the results from the multivariate analysis were recorded. Data extraction was completed by one investigator and checked by a second investigator for accuracy and consistency.

### Outcomes and data analysis

The primary aim of this systematic review was to determine the risk factors for mortality among neonates and infants hospitalized with an infectious process in developing countries. All risk estimates pertain to in-patient mortality, except those specifically noted as risk factors for outpatient (i.e., post-discharge) mortality.

As all studies were interpreted from a cohort design perspective, their quality was assessed using the Newcastle-Ottawa Scale (16). Each study was reviewed by one investigator (LE) for criteria related to selection, comparability, and outcome assessment.

Due to expected heterogeneity in study population, risk factors and analysis, it was determined *a priori* that formal meta-analysis would not be conducted. The primary form of analysis was therefore descriptive and conducted using Microsoft Excel (Redmond, WA). Studies were grouped according to both participant age group (neonatal vs. infant) and underlying population (i.e., disease etiology).

## Results

### Summary of included articles

**Figure 1:**
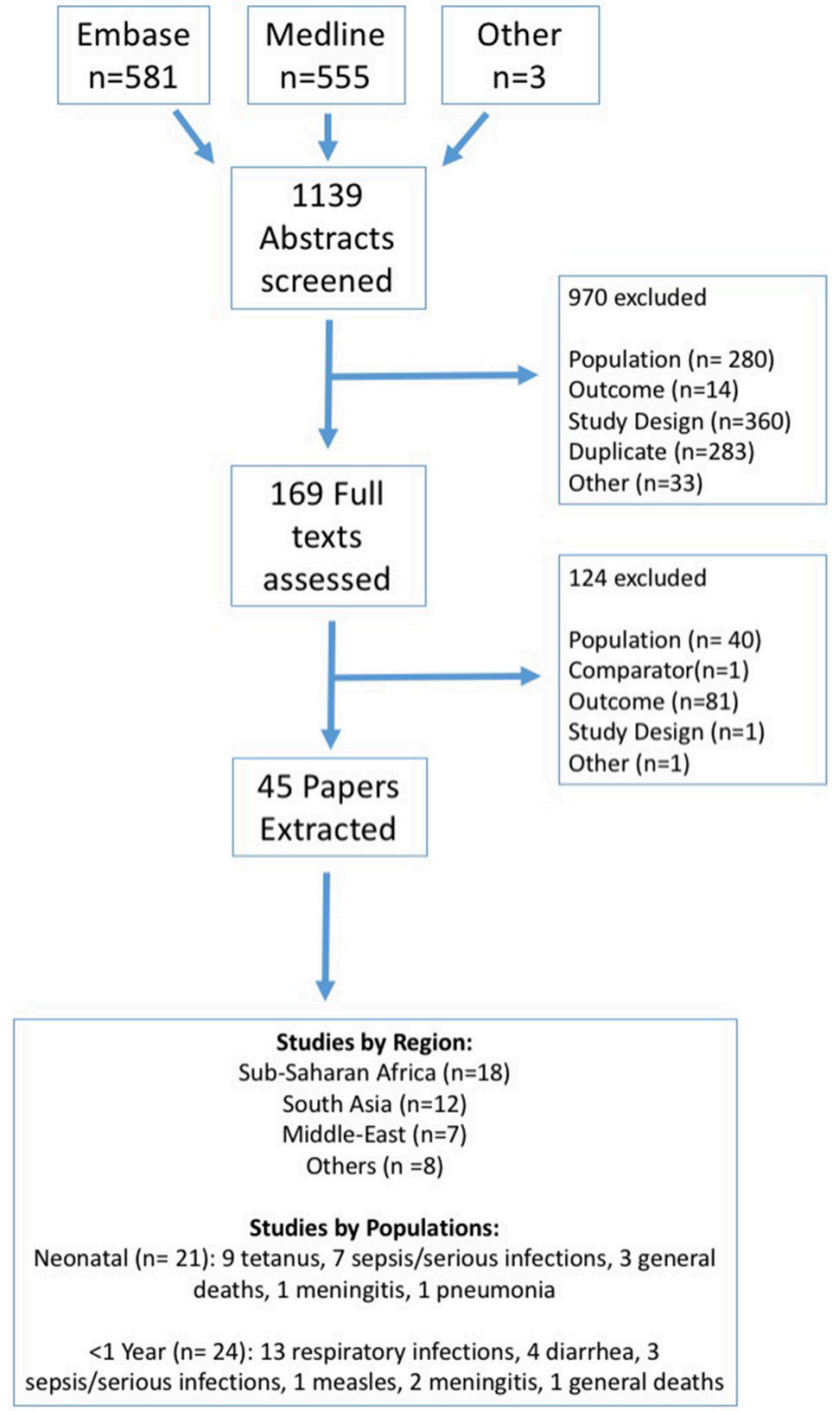
Systematic review flowchart.

Through the systematic literature search, 1,139 abstracts were identified. Of these, 970 were excluded during the abstract screening stage. The full-text of the remaining 169 articles were reviewed, after which an additional 124 articles were excluded. Thus, a total of 45 articles were included (Figure 1) (17–62).

Among these 45 studies, the majority of studies were prospective or retrospective cohort studies (Tables 3, 4). Upon assessment using the Newcastle-Ottawa scale, the average score for included studies was 6.3 out of 9 stars, based on selection criteria, comparability of cohorts, and assessment of outcomes encompassing attrition. Included studies were found to have low comparability, with only 40% controlling for confounders in the study design or statistical analysis. Approximately 70% of studies chose representative samples and reported reliable inclusion criteria.

Among the 45 included studies, multiple geographic regions were represented: 18 from Sub-Saharan Africa, 12 from South Asia, 7 from the Middle-East and 8 from other regions (Table 2). Twenty-one (46.6%) focused exclusively on the neonatal populations, whereas the other 24 studies (53.3%) focused on children under 1 year of age (Table 2). Nearly all of the studies (*n* = 42, 93.3%) focused exclusively on in-hospital deaths, while two (4.4%) studies focused exclusively on post-discharge death and one study (2.2%) on both in-hospital and post-discharge death. Seventeen (37.8%) of the included studies were published before 2000. The median number of study participants was 201 children (IQR 102-697).

**Table 2:** Study characteristics.

**Author and year**	**Study countries**	**Study period^a^**	**Study type**	**Study population**	**Mortality type (IP/PD)^b^**	**Total No. of patients**	**No. of patients who died**	**No. of patients < 1 month**	**No. of patients < 1 year**
**NEONATAL STUDIES (**<**1 MONTH)**									
Basu et al. (18)	India	1997–2003	Retrospective	Neonatal tetanus	IP + PD	101	67	101	101
Ballot et al. (17)	South Africa	2007–2011	Retrospective	Neonatal fungal sepsis	IP	63	27	63	63
Chiabi et al. (20)	Cameroon	2008–2009	Prospective	Neonatal sepsis	IP	218	46	218	218
Daoud et al. (22)	Jordan	1992–1994	Prospective	Neonatal meningitis	IP	53	17	53	53
Davies-Adetugbo et al. (23)	Nigeria	1991–1995	Retrospective	Neonatal tetanus	IP	174	96	174	174
Dikici et al. (25)	Turkey	1991–2006	Retrospective	Neonatal tetanus	IP	67	27	67	67
Ertem et al. (28)	Turkey	1994–2001	Retrospective	Neonatal tetanus	IP	56	38	56	56
Ghiorghis et al. (29)	Ethiopia	1992–1993	Retrospective	Neonatal sepsis	IP	542	195	542	542
Gurkan et al. (31)	Turkey	1991–1997	Retrospective	Neonatal tetanus	IP	55	22	55	55
Gurses et al. (32)	Turkey	1978–1988	Retrospective	Neonatal tetanus	IP	133	54	133	133
Ibinda et al. (33)	Kenya	1999–2013	Retrospective	Neonatal tetanus	IP	191	118	191	191
Mathur et al. (37)	India	NA	Prospective	Neonatal respiratory distress	IP	150	48	150	150
Mugalu et al. (40)	Uganda	2002	Prospective	Neonatal septicemia	IP	110	20	110	110
Okomo et al. (44)	Gambia	2009–2013	Retrospective	All neonatal deaths	IP	5,285	1,734	5,285	5,285
Okoromah et al. (45)	Nigeria	NA	Retrospective	Neonatal tetanus	IP	39	4	39	39
Ozkan et al. (46)	Turkey	2003–2010	Retrospective	Neonatal sepsis (preterm)	IP	151	22	151	NA
Sarna et al. (52)	India	1988	Retrospective	All neonatal deaths	IP	7,309	328	328	328
Saleem et al. (50)	Pakistan	2006–2011	Retrospective	Neonatal sepsis	IP	104	17	104	104
Sheikh et al. (55)	Pakistan	2012	Prospective	Neonatal sepsis	IP	125	20	125	125
Simiyu et al. (56)	Kenya	2000	Retrospective	All neonatal deaths	IP	308	97	308	308
Yaramis et al. (60)	Turkey	1990–1999	NA	Neonatal tetanus	IP	73	38	73	73
**INFANT STUDIES (**<**12 MONTHS)**									
Bhatnagar et al. (19)	India	2005–2008	RCT	Serious bacterial infection	IP	848	7	182	848
Coakley et al. (21)	Papua New Guinea	1989	Prospective	Measles	IP	282	48	NA	NA
Demers et al. (24)	Central African Republic	1996–1997	Prospective	Acute respiratory infections	IP	395	49	14	222
Djelantik et al. (26)	Indonesia	1999–2001	Retrospective	Pneumonia	IP	4,351	505	NA	NA
Duke et al. (27)	Papua New Guinea	1998–1999	Prospective	Pneumonia	IP	703	46	NA	NA
Goel et al. (30)	South Africa	1989–1995	Retrospective	Staphylococcal pneumonia	IP	100	7	NA	78
Islam et al. (62)	Bangladesh	1991–1992	Prospective	Diarrhea	PD	427	30	NA	329
Khan et al. (34)	Pakistan	2007–2008	Retrospective	Sepsis	IP	133	32	NA	NA
Kuti et al. (35)	Nigeria	2011–2013	Retrospective	Bacterial meningitis	IP	81	22	NA	NA
Lehmann et al. (36)	Papua New Guinea	1989–1992	Prospective	Bacterial meningitis	IP	696	96	NA	535
Moisi et al. (39)	Kenya	2004–2008	Prospective	General mortality	PD	14,971	535	NA	NA
Mulholland et al. (41)	Gambia	1990–1991	Prospective	Serious infections	IP	697	64	NA	697
Nantanda et al. (42)	Uganda	2005–2006	Prospective	Pneumonia	IP	157	24	87	NA
Nathoo et al. (43)	Zimbabwe	1989–1990	Prospective	Lower respiratory infection	IP	704	104	0	502
Ramakishna et al. (47)	Malawi	2005–2006	Prospective	Pneumonia	IP	233	25	0	117
Rodriguez et al. (48)	Columbia	2009–2011	Retrospective	Respiratory syncytial virus infection	IP	2,147	25	NA	1,549
Sachdev et al. (49)	India	1988	Prospective	Diarrhea	IP	382	37	NA	NA
Sehgal et al. (53)	India	1993–1994	Prospective	Lower respiratory infection	IP	201	21	NA	105
Santhanakrishnan et al. (51)	India	NA	NA	Diarrhea, acute	IP	575	64	NA	441
Shann et al. (54)	Papua New Guinea	NA	Prospective	Pneumonia	IP	47	7	NA	NA
Smyth et al. (57)	Zambia	1994–1995	Prospective	Pneumonia	IP	167	23	NA	104
Tupasi et al. (59)	Philippines	1981–1983	Prospective	ALRI	IP	726	34	NA	361
Teka et al. (58)	Bangladesh	1990–1994	Case-control	Diarrhea	IP	928	46	NA	NA
Zhang et al. (61)	China	2007–2010	Prospective	Pneumonia	IP	707	41	NA	535

a *All studies cohort unless otherwise specified*.

b *IP, inpatient; PD, post-discharge*.

Among 21 neonatal studies, nine focused on tetanus, seven on sepsis, one on meningitis, one on respiratory infections, and three on general admissions (Table 2). Among the 24 infant studies, 13 focused on children with respiratory infections. The remaining focused on diarrhea (*n* = 4), sepsis or serious infections (*n* = 3), meningitis (*n* = 2), measles (*n* = 1) and general admissions (*n* = 1; Table 2). Key results, along with definitions for all parameters, are highlighted in Tables 3, 4. For each illness, the most common risk factors, prioritized by frequency, are presented in these tables to ensure conciseness. Risk factors discussed only in a single study were not included in these tables, but are reported in the Supplementary Table 1.

**Table 3:** Summary of risk factors for neonatal mortality by underlying disease.

**Risk factor**	**Included study**	**Risk estimate^+^(95%CI)**	**Risk factor definitions**
**TETANUS (*****n*** = **9 STUDIES)**			
Cyanosis	Dikici et al. (25)	1.1 (0.2–7)	NR
	Gurkan et al. (31)	**NSS, estimate NR**	NR
	Gurses et al. (32)	**NSS, estimate NR**	NR
	Okoromah et al. (45)	**SS** > **1, estimate NR**	NR
Fever	Basu et al. (18)^*^	**4.6 (2.8–7.6)**	Not reported
	Ertem et al. (28)	**17.92 (2.29–135.53)**	NR
	Gurses et al. (32)	**SS** > **1, estimate NR**	NR
	Okoromah et al. (45)	**SS** > **1, estimate NR**	>40°C
	Yaramis et al. (60)	**SS** > **1, estimate NR**	NR
	Dikici et al. (23)	0.4 (0.4–5)	CV
	Gurkan et al. (31)	NSS, estimate NR	NR
Risus sardonicus	Basu et al. (18)^*^	**4.5 (2.4–8.6)**	NR
	Gurkan et al. (31)^*^	**SS** > **1, estimate NR**	NR
	Gurses et al. (32)	**SS** > **1, estimate NR**	NR
	Dikici et al. (23)	1.8 (0.1–20)	NR
Sex	Davies-Adetugbo et al. (23)	NSS, estimate NR	Male
	Dikici et al. (23)	1.2 (0.3–4)	Male
	Ibinda et al. (33)	0.67 (0.72–1.25)	Male
	Gurses et al. (32)	NSS, estimate NR	Male
	Okoromah et al. (45)	NSS, estimate NR	Male
Tetanic spasms, spasticity	Basu et al. (18)^*^	0.9 (0.5–1.9)	NR
	Dikici et al. (23)	1.1 (0.2–7)	NR
	Gurkan et al. (31)	NSS, estimate NR	NR
	Ibinda et al. (33)	**1.15 (1.04–1.26)**	NR
Young age	Davies-Adetugbo et al. (23)	**3.11 (1.04–5.8)**	< 6 days
	Dikici et al. (23)	**5.28 (1.73–16.165)**	NR
	Gurkan et al. (31)	**SS** > **1, estimate NR**	NR
	Gurses et al. (32)	**SS** > **1, estimate NR**	NR
	Ibinda et al. (33)	**1.64 (1.22–2.18)**	NR
	Okoromah et al. (45)	NSS, estimate NR	NR
Young age at onset of symptoms	Gurses et al. (32)	**SS** > **1, estimate NR**	NR
	Yaramis et al. (60)	**SS** > **1, estimate NR**	NR
	Dikici et al. (23)	0.6 (0.3–1)	CV
	Ertem et al. (28)	0.6 (0.55–1)	CV
	Okoromah et al. (45)	NSS, estimate NR	NR
**NEONATAL SEPSIS/SEPTICEMIA (*****n*** = **6 STUDIES)**			
Gender	Sheikh et al. (55)	**SS** > **1, estimate NR**	Male
	Saleem et al. (50)	3 (0.9–9.5)	Male
Late onset of sepsis	Ozkan et al. (46)	**1.2 (1–2.7)**	Late onset: 3–30 days
	Sheikh et al. (55)	**SS**<**1, estimate NR**	Late onset: 3–30 days
Low birthweight	Chiabi et al. (20)	**SS** > **1, estimate NR**	< 2,500 g
	Ghiorghis et al. (29)	NSS, estimate NR	NR
	Saleem et al. (50)	6.1 (0.8–44.4)	< 1,000 g
Positive blood culture	Chiabi et al. (20)	NSS, estimate NR	NR
	Sheikh et al. (55)	**SS** > **1, estimate NR**	NR
Prematurity/low gestation age	Chiabi et al. (20)	NSS, estimate NR	< 37 weeks
	Ghiorghis et al. (29)	**2.22 (1.07–4.63)**	< 38 weeks
	Sarna et al. (52)	NSS, estimate NR	CV
**GENERAL DEATHS (*****n*** = **3 STUDIES)**			
Hypothermia	Okomo et al. (44)	**2.48 (1.76–3.49)**	< 36.5°C
	Simiyu et al. (56)	**7.14 (2.22–40)**	< 36.5°C
Lack of maternal antenatal care	Okomo et al. (44)	**1.68 (1.17–2.41)**	NA
Low admission weight	Okomo et al. (44)	**1.61 (1.15–2.26)**	< 1,500 g
	Simiyu et al. (56)	**5.88 (3.03–11.1)**	< 2,500 g
Low birthweight	Sarna et al. (52)	**SS** > **1, estimate NR**	< 2,000 g
Prematurity, low gestation age	Sarna et al. (52)	**SS** > **1, estimate NR**	< 37 weeks

*All risk estimates are odds ratios, unless relative risk ratio denoted by RR*.

*Bolded risk factors are statistically significant*.

+ *Denotes all risk estimates are odds ratios, unless relative risk ratio denoted by RR*.

* *Denotes that post-discharge populations were assessed*.

*SS>1, statistically significant association with risk estimate>1*.

NR denotes “Not Reported.”

NA denotes “Not Applicable.”

*NSS denotes not statistically significant*.

**Table 4:** Summary of risk factors for infant mortality by underlying disease.

**Risk factor**	**Included study**	**Risk estimate^+^(95%CI)**	**Risk factor definitions**
**DIARRHEA (*****n*** = **4 STUDIES)**			
Dehydration	Sachdev et al. (49)	1.96 (0.5–8.2)	Severe dehydration
	Islam et al. (62)^*^	NSS, estimate NR	Severe dehydration
Maternal education	Islam et al. (62)^*^	**2.12 (1.37–3.28)**	< 1 year of schooling
	Teka et al. (58)	1.96 (0.5–0.82)	No education
Non-breastfed	Santhanakrishnan et al. (51)	**2.29, estimate NR**	Not exclusively breastfed
	Teka et al. (58)	**4.19 (1.3–13.2)**	Non-breastfed
	Islam et al. (62)^*^	**2.35 (1.44–3.84)**	Non-breastfed
Poor nutritional status	Sachdev et al. (49)	**3.3 (2.7–4)**	Weight for age < 50%
	Sachdev et al. (49)	**1.9 (1.6–23)**	Height for age < 85%
	Santhanakrishnan et al. (51)	**4.94, estimate NR**	Degree of malnutrition of 2 or 3 on Gomez scale
	Teka et al. (58)	**84.16 (9.1–775.9)**	Weight for height < 70%
	Islam et al. (62)^*^	**2.97 (1.43–6.16)**	Height for age < 85%
Young age	Islam et al. (62)^*^	**4.57 (2.9–7.18)**	Age < 6 months
	Santhanakrishnan et al. (51)	NSS, estimate NR	Age < 6 months
	Sachdev et al. (49)	NSS, estimate NR	Age < 6 months
**ACUTE RESPIRATORY INFECTION OR PNEUMONIA (*****n*** = **13 STUDIES)**			
Alteration of status	Demers et al. (24)	**3.23 (1.17–8.94)**	Moderate/severe mental alteration
	Sehgal et al. (53)	NSS, estimate NR	NR
	Sehgal et al. (53)	NSS, estimate NR	NR
	Zhang et al. (61)	NSS, estimate NR	NR
Cyanosis	Zhang et al. (61)	NSS, estimate NR	Central cyanosis
	Duke et al. (27)	1.46 (0.79–3.45)	NR
	Sehgal et al. (53)	NSS, estimate NR	NR
	Nantada et al. (42)	NSS, estimate NR	NR
Low oxygen saturation	Djelantik et al. (26)	**2.7 (2.1–3.5)**	SpO_2_ < 85%
	Duke et al. (27)	**2.46 (1.3–4.65)**	SpO_2_ < 70%
	Nantanda et al. (42)	**SS** > **1, estimate NR**	NR
	Smyth et al. (57)	**SS** > **1, estimate NR**	NR
	Ramakishna et al. (47)	**2.96 (0.94–0.29)**	SpO_2_ < 90%
	Zhang et al. (61)	NSS, estimate NR	SpO_2_ < 90%
Low respiratory rate	Djelantik et al. (26)	**4.6 (2.5–8.5)**	< 40 breaths per minute
	Shann et al. (54)	NSS, estimate NR	< 40 breaths per minute
Malnutrition	Djelantik et al. (26)	1.1(0.93–1.3)	Weight for age < 5th percentile
	Demers et al. (24)	**2.74 (0.96–7.76)**	Weight for height >2SD less than median for age
	Nantanda et al. (42)	**SS** > **1, estimate NR**	NR
	Nathoo et al. (43)	**3.8 (2.7–5.4)**	Weight for age < 60%
	Sehgal et al. (53)^*^	**3.9 (1.01–9.07)**	WAZ < −3
	Tupasi et al. (59)	**4.4 (2–9.52)**	First degree malnutrition on Gomez Scale
	Duke et al. (27)	**6.32 (2.74–14.57)**	Weight for age < 60%
	Smyth et al. (57)	**SS** > **1, estimate NR**	Low WAZ^&^
	Shann et al. (54)	NSS, estimate NR	Weight for age < 80%
Young age	Djelantik et al. (26)	**3.6 (2.8–4.6)**	Age < 4 months
	Nathoo et al. (43)	**2.4 (1.6–3.5)**	Age < 6 months
	Rodriguez et al. (48)	1.11(0.51–2.41)	Age < 6 months
	Smyth et al. (57)	NSS, estimate NR	Age < 6 months
**SERIOUS INFECTION OR SEPSIS (*****n*** = **3 STUDIES)**			
Bacteremia	Mulholland et al. (41)	**SS** > **1, estimate NR**	NR
Need for >2 inotropes	Khan et al. (34)	**3.5 (1.3–9.2)**	>2 inotropes
Organ dysfunction	Khan et al. (34)	**18 (2.2–144)**	>2 dysfunctional organs
PRISM score >10	Bhatnagar et al. (19)^Intervention^	0.57 (0.27–1.23)	10 mg zinc intake daily
Zinc intake	Khan et al. (34)	1.5 (0.6–4)	>10

* *Denotes that post-discharge populations were assessed*.

*Bolded risk factors are statistically significant*.

+ *Denotes all risk estimates are odds ratios, unless relative risk ratio denoted by RR*.

& *Denotes low weight-for-age Z score*.

NR denotes “Not Reported.”

NA denotes “Not Applicable.”

*SS>1, statistically significant association with risk estimate >1*.

*SS < 1, statistically significant association with risk estimate < 1*.

**Intervention:** *In this study, the intervention was zinc intake and the comparator was normal care. The exposure (suspected bacterial infection) was the same for both groups. In all other studies included in this systematic review, the intervention is usual care with exposure to the risk factor, and the comparator is intervention without exposure to risk factor*.

### Neonatal mortality

#### Tetanus

A total of nine studies evaluated predictors of mortality for neonatal tetanus. Mortality rates ranged from 10.3 to 67.9% in these studies. The most consistent predictors of mortality were young age of presentation and onset, presence of fever and risus sardonicus. For young age at presentation, the adjusted odds ratios ranged from 1.64 (95% CI: 1.22–2.18) to 5.29 (95% CI: 1.73–16.17). The presence of fever was associated with increased odds of mortality, ranging from 4.6 (95% CI: 2.8–7.6) to 17.92 (95% CI: 2.29–135.53). Three of the five studies reported fever as a statistically significant variable, but did not provide odds ratios. The odds of mortality among patients with risus sardonicus study were 4.5 (95% CI: 2.4–8.6) in one study. Two studies also found risus sardonicus and young age of onset to be significantly associated with mortality, although specific values were not provided.

Variables frequently assessed as potential risk factors, but which consistently showed no association with mortality in this population included sex, tetanic spasms/spasticity, umbilical inflammation, sepsis, trismus, maternal tetanus immunization status, lack of sucking, omphalitis, irritability, high heart rate and high respiratory rate.

#### Sepsis/serious infections

Seven studies evaluated mortality among populations of neonates with sepsis or severe infection. Mortality rates were very high, ranging from 14.6 to 36.0% of admitted patients. Prematurity and low birth weight were significantly associated with mortality in two studies for each of these factors. The odds ratio for prematurity was 2.22 (95% CI: 1.07–4.63) for one study and not given for another study. Similarly, the odds ratio for low birth weight was 6.1 (95% CI: 0.8–44.4) in one study and not given for another study. Platelet count, weight at presentation, sex and white blood cell counts did not show statistically significant correlations with mortality in these studies.

#### General hospital admissions

Three studies assessed mortality among general neonatal hospital admissions. These studies have included neonatal populations with diarrhea, malaria, pneumonia, jaundice, convulsions, soft-tissue infections, asphyxia, tetanus, omphaitis, congenital malformations, intrapartum abnormalities and hypothermia. Low admission weight and hypothermia were found to be statistically significant predictors of mortality in both studies in which they were assessed. The odds of mortality for children with low admission weight was 1.61 (95% CI: 1.15–2.26) and 5.88 (95% CI: 3.03–11.1), and for children with hypothermia was 2.48 (95% CI: 1.76–3.49) and 7.14 (95% CI: 2.22–40). Maternal age, sex and time of day at presentation were not significant correlates of mortality in these studies.

#### Other studies

In the single study assessing mortality among neonates with meningitis, a bulging anterior fontanelle was the only significant predictor of mortality, with an odds ratio of 7.7 (95% CI: 1.7–35.4). Hypothermia, feeding difficulties, jaundice, cyanosis, vomiting, convulsions and respiratory distress showed no significant correlation with mortality among meningitis patients. It is worth mentioning that the above study only contained a total of 17 deaths, so was likely underpowered. In the single study focused on respiratory infections among neonates, an alveolar-arterial carbon dioxide gradient (AaCO2 gradient) >250 mmHg was the only statistically significant predictor of mortality, with an odds ratio of 71.1 (95% CI: 1.1–4,395). Weight, gestational age, lethargy, age at presentation, pH < 7.2, absent neonatal reflexes, shock, fraction of inspired oxygen (FiO2), blood base excess, blood culture, C-reactive protein level, arterial alveolar oxygen ratio, and ventilatory support showed little or no correlation with mortality among patients with respiratory infections. Many of these variables were significant correlates of mortality in univariate analysis but lost significance in multivariate analysis. However, it is possible that the study is underpowered since only a total of 22 deaths were reported in the study.

### Infant (< 1 year) mortality

#### Respiratory infections

In the 13 studies evaluating respiratory tract infections, mortality rates ranged from 1.2 to 15.3% among admitted infants. Malnutrition, low oxygen saturation, younger age and positive Human Immunodeficiency Virus (HIV) status were top predictors of mortality in these patients. The odds of mortality among malnourished patients varied from 3.8 (95% CI: 2.7–5.4) to 6.32 (95% CI: 2.72–14.57). Among four studies, the odds of mortality among patients with lower oxygen saturation were 2.7 (95% CI: 2.1–3.5), 2.46 (95% CI: 1.3–4.65), and significant, but not provided, in the two remaining studies. The odds ratios of mortality among younger infants with respiratory infections, defined as < 4 months in one study and < 6 months in another, varied from 2.4 (95% CI: 1.6–3.5) to 3.6 (95% CI: 2.8–4.6). One study showed an association between high respiratory rate and mortality, while another showed high respiratory rates to be significantly associated with mortality. Cyanosis (four studies), wheezing (four studies), duration of breastfeeding (two studies) and congenital cardiac abnormalities (two studies) were not found to be correlates of mortality in these studies.

#### Diarrhea

In the four studies focused on infants admitted with diarrhea, the mortality rate was between 5 and 11.1%. Malnutrition and the absence of breastfeeding were the top predictors of mortality among both in-hospital and post-discharge populations, showing statistically significant correlations with mortality. The odds of mortality varied widely from 1.9 (95% CI: 1.6–2.3) to 84.16 (95% CI: 9.1–775.9) among malnourished patients, and from 2.35 (95% CI: 1.44–3.84) to 4.2 (95% CI: 1.3–13.2) among non-breastfed patients. Dehydration status, blood in stools, xerophthalmia, and concurrent pneumonia did not appear as statistically significant correlates of mortality.

#### Sepsis/serious infections

In the three studies focused on sepsis or serious infection, organ dysfunction, the need for more than 2 inotropes and bacterial isolates in blood showed significant correlations with mortality. The odds of mortality was 18 (95% CI 2.2–144) for organ dysfunction, 3.5 (95% CI 1.3–9.2) for those needing for more than 2 inotropes and significant, although not specifically provided, for those with bacteremia. Zinc intake and a Pediatric Risk of Mortality (PRISM) score >10 were not statistically significantly correlated in these studies.

#### Other studies

Two studies assessed predictors of mortality among patients with meningitis. Low hemoglobin (OR 2.9, 95% CI: 1.5–5.3), refusal or inability to feed (OR 3.3, 95% CI: 2.1–5.6), vomiting (OR 1.7, 95% CI: 1.2–2.7), and drowsiness (OR 2.9, 95% CI: 1.7–5) were significant predictors of mortality. In addition, high WBC count and low CSF glucose were significantly associated with increased odds of mortality, although odds ratios were not specifically stated. Multiple seizures, coma at presentation, neck stiffness, hyponatremia, hyperglycemia, hypoglycorrhachia, and turbid CSF showed no significant correlation with mortality. This is likely underpowered as only 22 deaths were reported in the study describing the above risk factors.

One study focused on mortality among a post-discharge population. This study found malnutrition defined as weight-for-age Z score < −3 (OR 3.42, 95% CI 2.5–4.68), hypoxia (OR 2.3, 95% CI: 1.64–3.23), bacteremia (OR 1.77, 95% CI: 1.15–2.74), jaundice (OR 1.77, 95% CI: 1.08–2.91), hepatomegaly (OR 2.34, 95% CI: 1.6–3.42), and hospitalization length < 13 days (OR 1.83, 95% CI: 1.33–2.52) showed significant correlations with post-discharge mortality. Age and parasitemia showed no correlation with mortality outcomes.

Lastly, in one study on infants with measles, low birth weight and nosocomial infections showed significant correlations with mortality, although the odds ratios were not provided. Infant age and vitamin A supplementation did not correlate with mortality.

## Discussion

This systematic review focused on risk factors of mortality among hospitalized neonates and infants under 1 year of age due to infectious causes in LMICs. To the best of our knowledge, this is the first systematic literature review that focuses of clinical predictors of mortality from severe infection among hospitalized neonates and infants in developing countries. Most previous work on these populations consist of single population-based studies (11, 63). Previous systematic reviews of clinical predictors of mortality from severe infections have included infants in developed countries, which may not applicable to areas of the world where the majority of the disease burden occurs (10). Furthermore, other systematic reviews have examined predictors of severe illness and/hospitalization rather than mortality (64, 65), the latter which is a more objective, directly actionable outcome for use in intervention trials. Moreover, other previous work have focused on on all-cause mortality from sepsis as universally preventable disease, rather than more specific illness populations (7). This evidence base is complementary to publications from the WHO, CDC, and other international organizations, which approach neonatal and infant mortality from a population perspective. This systematic review consolidates mortality outcomes on the individual and community level, by focusing on clinical, laboratory and socio-demographic risk factors. It can be used to guide interventions to address the epidemic of early mortality among over 4 million infants and neonates in developing countries.

Comprehensive understanding of the clinical risk factors for mortality in these populations is important for several reasons. First, compilation of risk factors will help to drive the development risk prediction models aimed at the identification of high-risk infants, in order to triage resources to improve health outcomes and health care efficiency. This precision public-health approach has previously been utilized to derive risk-prediction models. For example, the miniPIERS, a prediction model to identify women at risk of hypertensive-related death, was developed to intervene among high-risk pregnant women in LMICs (66). Similar models have been created to identify high-risk patients with Chagas disease and to predict pediatric post-discharge mortality (67–69). Second, expanding our understanding of clinical risk factors for mortality in this age group could highlight key diagnostic approaches and research gaps in these areas. Lastly, specific populations or diseases may have limited research, and the benefits of systematic reviews in the identification of such gaps is well-described (12, 13).

The predictors of mortality were assessed for two distinct age groups: neonates (< 28 days old) and infants (28 days to 12 months old). Overall, the most frequent predictors of mortality in neonates were young age, fever or hypothermia, low birth weight and prematurity, while the most frequent predictors of mortality in infants were malnutrition, breast-feeding status and low oxygen saturation. It appears that perinatal variables play a larger role in predicting mortality during the neonatal period, whereas nutritional factors play a larger role among infants. Although both age groups encompassed a variety of disease populations, these risk factors found commonality in predicting mortality despite the heterogeneity in underlying conditions. Some of these predictors, such as temperature instability, young age, low birth weight, and prematurity, may not be surprising from a diagnostic perspective, as they are directly related to inherent causes of neonatal mortality. However, other predictors, such as a low oxygen saturation and breastfeeding, are more interesting from an interventional point of view. For instance, our results suggest that substantial efforts should be directed toward reducing malnutrition, promoting breastfeeding (parallel to HIV prevention) and developing affordable technologies for identifying oxygenation status among neonates and infants.

On the whole, included studies in this systematic review upheld strong research designs and maintained internal validity, based on the Newcastle-Ottawa scale. The primary limitation was comparability, as many studies did not adequately control for confounders, possibly due to small sample sizes. Retrospective studies utilizing medical record review were often faced with incomplete records, and the included studies may or may not have addressed this issue adequately. Other limitations of cohort studies often include recall and reporting bias, which were not assessed by this scale.

It is relevant to note that many of the studies identified for full text review assessed risk factors for serious illness, rather than mortality, and these studies were ultimately excluded (11, 70, 71). This systematic review focused primarily on predictors of mortality, since this downstream outcome is critical to the development of prediction tools or interventions to improve child health and survival. Furthermore, the definition of mortality is more consistent as compared to serious illness, which can have broad and heterogeneous definitions.

Although the definition of mortality is more objective, a major source of heterogeneity between studies was the variable definitions of risk factors. For example, malnutrition definitions varied from weight-for-age Z score < 3 standard deviations to weight-for-age Z score < 60% to first degree malnutrition on the Gomez scale (corresponding to < 90% expected weight-for-age). Likewise, definitions for low oxygen saturation varied substantially between studies. Low oxygen saturation ranged from SpO_2_ < 70 to < 92%. This heterogeneity was pervasive among sub-populations and limited the ability to perform meta-analyses. A further source of heterogeneity is the differences in inclusion criteria for populations with the same illness. For example, one study on if late-onset sepsis (defined as 3–30 days of life) is a risk factor for mortality looked at pre-term neonates whereas another study looked at at-term ones. This difference may be partially responsible for one studying showing a statistically significant positive correlation with mortality whereas the other showed a statistically significant negative correlation.

Although uncommon, a small proportion of included studies did not provide definition for the evaluated risk factors, such as fever and young age. The provision of clear definitions for risk factors is crucial for generalizability and study replication. Moreover, increased standardization of these definitions would allow future data to be more amenable to meta-analyses. This would greatly enhance the translation potential of these studies, especially since many of the existing individual studies are underpowered for statistical analysis.

This systematic review has several limitations. First, some studies identified in this review only conducted univariate analyses while others conducted multivariate analyses. Thus, risk factors identified as statistically significant in the univariate analyses may cease to be statistically significant if multivariate analyses were performed. Second, most studies focus on single disease populations, resulting in risk factors that may not be generalizable across multiple illness populations. Since sepsis is associated with most disease-related deaths (72), it may provide an overarching framework within which to assess mortality predictors that are applicable across different acute illness processes. Third, few studies focused on post-discharge mortality, resulting in a critical knowledge gap in child mortality research. Post-discharge mortality rates have been shown to be equal or greater than in-hospital mortality rates in pediatric populations (67). More post-discharge studies on neonates and infants would be useful in determining if there are additional risk factors relevant to this population. Fourth, many risk factors which we evaluated did not demonstrate statistical significance. Frequently, small sample sizes may have limited the ability to evaluate the association of important variables on mortality. These results underscore the need for adequately powered studies, but also clearly demonstrate the importance of those variables which did achieve statistical significance. Furthermore, some clinically important risk factors, such as maternal HIV status, maternal malnutrition and implementation of TB or HIV preventative therapy during pregnancy, may have not been captured as statistically significant risk factors as a result of many included studies being underpowered as well as the focus on hospitalized patients as an inclusion criteria. Lastly, many parameters found to be important predictors of mortality among infants, such as hemoglobin level, oxygen saturation and nutritional status, were not assessed in studies focused on serious infections among neonatal patients. The inclusion of these parameters in future studies has the potential to elucidate strong predictors of infant mortality.

## Conclusions

This systematic review summarizes risk factors for mortality among neonates and infants in LMICs. Our data highlight major risk factors that could be incorporated into risk-prediction models to identify children at risk for in-hospital or post-discharge mortality. This data also points toward specific interventions that could be further incorporated into healthcare systems or policies. Targeted, evidence-based interventions have the potential to vastly reduce the burden of preventable mortality among neonates and infants around the world. Future studies in this area should incorporate precise definitions and risk estimates for mortality, including larger sample sizes, detailed statistical analysis, and overlapping risk factors between these high-risk age groups.

## Author contributions

LL prepared first draft of manuscript, conducted systematic search and data extraction, assisted in analysis of data, approved final version of manuscript. NaK and LE conducted systematic search and data extraction, assisted in analysis of data, approved final version of manuscript. NiK, PL, JA, and JK reviewed and edited manuscript and provided critical interpretation of data, assisted in analysis of data, approved final version of manuscript. MW conceived study idea, assisted in systematic search and data extraction, assisted in analysis of data, reviewed and edited manuscript and provided critical interpretation of data, approved final version of manuscript.

### Conflict of interest statement

The authors declare that the research was conducted in the absence of any commercial or financial relationships that could be construed as a potential conflict of interest.
