# Supplementary material for: Predictors of Mortality in Neonates and Infants Hospitalized With Sepsis or Serious Infections in Developing Countries: A Systematic Review
Source: Front Pediatr. 2018 Oct 4;6:277. doi: 10.3389/fped.2018.00277 (PMC6190846; doi:10.3389/fped.2018.00277)
Supplement: Supplementary file 2 [file Data_Sheet_1.docx]

Appendix 1. Medline Search strategy

| **Medline Search - conducted April 14, 2017** | | |
| --- | --- | --- |
| **Item** | **Terms** | **Hits** |
| 1 | infection/ or communicable diseases/ or community-acquired infections/ or opportunistic infections/ or respiratory tract infections/ or sepsis/ or skin diseases, infectious/ or soft tissue infections/ or toxemia/ or urinary tract infections/ or exp bacterial infections/ or bacterial infections.ti,ab. or exp Pneumonia/ or pneumonia.ti,ab. or exp Meningococcal Infections/ or exp Sepsis/ or sepsis.ti,ab. or exp Malaria/ or malaria.ti,ab. or exp Diarrhea/ or exp Diarrhea, Infantile/ or diarrhea.ti,ab. or severe illness.ti,ab. | 1278300 |
| 2 | predict$.ti,ab. or exp Protective Factors/ or exp Risk Factors/ or (signs or symptoms or predictor* or risk factor* or clinical feature*).ti,ab. | 2821365 |
| 3 | exp Developing Countries/ or developing countries.ti,ab. or low resource countr$.ti,ab. or resource poor countr$.ti,ab. | 100482 |
| 4 | exp Infant/ or exp Infant, Newborn/ or neonat$.ti,ab. or infant$.ti,ab. | 1237205 |
| 5 | exp Mortality/ or mortality.ti,ab. or post discharge mortality.ti,ab. or community mortality.ti,ab. or long term mortality.ti,ab. or post hospital mortality.ti,ab. | 814363 |
| 6 | and/1-5 | 555 |

Appendix 2. Embase search strategy

| **EMBASE Search - conducted April 14, 2017** | | |
| --- | --- | --- |
| **Item** | **Terms** | **Hits** |
| 1 | infection/ or communicable disease/ or community-acquired infection/ or opportunistic infection/ or respiratory tract infection/ or exp sepsis/ or skin infection/ or toxemia/ or urinary tract infection/ or exp bacterial infection/ or Pneumonia/ or meningococcosis/ or exp Malaria/ or exp Diarrhea/ or bacterial infections.ti,ab. or pneumonia.ti,ab. or sepsis.ti,ab. or malaria.ti,ab. or diarrhea.ti,ab. or severe illness.ti,ab. | 1954045 |
| 2 | predict$.ti,ab. or exp Risk Factor/ or (signs or symptoms or predictor* or risk factor* or clinical feature*).ti,ab. | 3677006 |
| 3 | exp Developing Country/ or developing countr$.ti,ab. or low resource countr$.ti,ab. or resource poor countr$.ti,ab. | 120440 |
| 4 | Infant/ or Newborn/ or neonat$.ti,ab. or infant$.ti,ab. | 1193374 |
| 5 | exp Mortality/ or mortality.ti,ab. or post discharge mortality.ti,ab. or community mortality.ti,ab. or long term mortality.ti,ab. or post hospital mortality.ti,ab. | 1170678 |
| 6 | and/1-5 | 581 |

Appendix 3. Newcastle Ottawa Scale

| Author | Study Type | Selection | | | | Comparability | Outcome | | | Total |
| --- | --- | --- | --- | --- | --- | --- | --- | --- | --- | --- |
|  |  | Representative exposed | Selection unexposed | Exposure ascertainment | Temporality | Controls | Outcome assessment | Adequate length of follow-up | Minimal attrition |  |
| Neonatal Studies | | | | | | | | | | |
| Basu et al, 2006 ^[16]^ | Retrospective | 1 | 1 | 1 | 1 |  | 1 | 1 |  | 6 |
| Ballot et al, 2013 ^[15]^ | Retrospective | 1 | 1 | 1 | 1 |  | 1 | 1 | 1 | 7 |
| Chiabi et al, 2011 ^[18]^ | Prospective | 1 | 1 | 1 | 1 |  |  | 1 |  | 5 |
| Daoud et al, 1996 ^[20]^ | Prospective | 1 | 1 | 1 | 1 | 1 |  | 1 | 1 | 7 |
| Davies-Adetugbo et al, 1998 ^[21]^ | Retrospective | 1 | 1 | 1 | 1 |  | 1 | 1 | 1 | 7 |
| Dikici et al, 2008 ^[23]^ | Retrospective | 1 | 1 | 1 | 1 |  | 1 | 1 | 1 | 7 |
| Ertem et al, 2004 ^[26]^ | Retrospective |  | 1 | 1 | 1 | 1 | 1 |  | 1 | 6 |
| Ghiorghis et al, 1997 ^[27]^ | Retrospective | 1 | 1 | 1 | 1 |  | 1 | 1 | 1 | 7 |
| Gurkan et al, 1999 ^[29]^ | Retrospective | 1 | 1 | 1 | 1 |  | 1 | 1 | 1 | 7 |
| Gurses et al, 1993 ^[30]^ | Retrospective | 1 | 1 |  | 1 |  | 1 | 1 | 1 | 6 |
| Ibinda et al, 2015 ^[31]^ | Retrospective | 1 | 1 | 1 | 1 | 1 | 1 | 1 | 1 | 8 |
| Mathur et al, 2002 ^[35]^ | Prospective | 1 | 1 | 1 | 1 | 1 | 1 | 1 | 1 | 8 |
| Mugalu et al, 2006 ^[38]^ | Prospective | 1 |  | 1 | 1 |  | 1 | 1 | 1 | 6 |
| Okomo et al, 2015 ^[42]^ | Retrospective |  | 1 |  | 1 | 1 | 1 | 1 | 1 | 6 |
| Okoromah et al, 2003 ^[43]^ | Retrospective | 1 | 1 |  | 1 |  | 1 | 1 | 1 | 6 |
| Ozkan et al, 2013 ^[44]^ | Retrospective |  | 1 | 1 | 1 |  | 1 | 1 | 1 | 6 |
| Sarna et al, 1991 ^[50]^ | Retrospective | 1 | 1 |  | 1 |  | 1 | 1 | 1 | 6 |
| Saleem et al, 2013 ^[49]^ | Retrospective | 1 | 1 |  | 1 | 1 | 1 | 1 | 1 | 7 |
| Sheikh et al, 2014 ^[53]^ | Prospective |  | 1 |  | 1 |  | 1 | 1 | 1 | 5 |
| Simiyu et al, 2003 ^[54]^ | Retrospective | 1 | 1 | 1 | 1 |  | 1 | 1 | 1 | 7 |
| Yaramis et al, 2000 ^[58]^ | Retrospective | 1 | 1 |  | 1 |  | 1 | 1 | 1 | 6 |
| Infant Studies | | | | | | | | | | |
| Bhatnagar et al, 2012 ^[17]^ | RCT | 1 | 1 |  | 1 | 2 | 1 |  | 1 | 7 |
| Coakley et al, 1991 ^[19]^ | Prospective | 1 | 1 |  | 1 |  | 1 |  | 1 | 5 |
| Demers et al, 2000 ^[22]^ | Prospective | 1 | 1 |  | 1 | 1 | 1 | 1 |  | 6 |
| Djelantik et al, 2003 ^[24]^ | Retrospective |  | 1 | 1 | 1 | 1 | 1 | 1 | 1 | 7 |
| Duke et al, 2001 ^[25]^ | Prospective | 1 |  | 1 | 1 | 1 | 1 | 1 | 1 | 7 |
| Goel et al, 1999 ^[28]^ | Retrospective | 1 | 1 | 1 | 1 |  |  | 1 | 1 | 6 |
| Islam et al, 1996 ^[60]^ | Prospective | 1 | 1 | 1 | 1 |  | 1 | 1 |  | 6 |
| Khan et al, 2012 ^[32]^ | Retrospective |  | 1 | 1 | 1 |  | 1 | 1 | 1 | 6 |
| Kuti et al, 2015 ^[33]^ | Retrospective |  | 1 | 1 | 1 | 1 | 1 | 1 | 1 | 7 |
| Lehmann et al, 1999 ^[34]^ | Prospective |  | 1 | 1 | 1 |  | 1 | 1 | 1 | 6 |
| Moisi et al, 2011 ^[37]^ | Prospective | 1 | 1 | 1 | 1 | 1 | 1 | 1 | 1 | 8 |
| Mulholland et al, 1999 ^[39]^ | Prospective |  | 1 | 1 | 1 |  | 1 | 1 | 1 | 6 |
| Nantanda et al, 2008 ^[40]^ | Prospective | 1 | 1 | 1 | 1 | 1 | 1 | 1 | 1 | 8 |
| Nathoo et al, 1993 ^[41]^ | Prospective | 1 | 1 |  | 1 |  | 1 | 1 | 1 | 6 |
| Ramakishna et al, 2012 ^[45]^ | Prospective |  | 1 | 1 | 1 | 1 | 1 | 1 | 1 | 7 |
| Rodriguez et al, 2014 ^[46]^ | Retrospective |  | 1 | 1 | 1 |  | 1 | 1 | 1 | 6 |
| Sachdev et al, 1991 ^[47]^ | Prospective | 1 | 1 | 1 | 1 | 1 | 1 | 1 | 1 | 8 |
| Sehgal et al, 1997 ^[51]^ | Prospective | 1 | 1 | 1 | 1 |  | 1 | 1 | 1 | 7 |
| Santhanakrishnan et al, 1987 ^[49]^ | Prospective |  | 1 | 1 | 1 |  | 1 | 1 | 1 | 6 |
| Shann et al, 1998 ^[52]^ | Prospective |  | 1 |  | 1 |  | 1 | 1 | 1 | 5 |
| Smyth et al, 1998 ^[55]^ | Prospective | 1 | 1 |  | 1 |  | 1 | 1 | 1 | 6 |
| Tupasi et al, 1988 ^[57]^ | Prospective | 1 | 1 |  | 1 | 1 | 1 |  | 1 | 6 |
| Teka et al, 1996 ^[56]^ | Case-control | 1 |  | 1 | 1 | 1 | 1 | 1 | 1 | 7 |
| Zhang et al, 2013 ^[59]^ | Prospective | 1 | 1 | 1 | 1 |  | 1 | 1 |  | 6 |
| **Percent Attained** | | 71% | 93% | 69% | 100% | 40% | 93% | 91% | 89% |  |
